# The Effects of Combinations of Cognitive Impairment and Pre-frailty on Adverse Outcomes from a Prospective Community-Based Cohort Study of Older Chinese People

**DOI:** 10.3389/fmed.2018.00050

**Published:** 2018-03-06

**Authors:** Ruby Yu, John E. Morley, Timothy Kwok, Jason Leung, Osbert Cheung, Jean Woo

**Affiliations:** ^1^Department of Medicine and Therapeutics, Faculty of Medicine, The Chinese University of Hong Kong, Shatin, Hong Kong; ^2^The Chinese University of Hong Kong Jockey Club Institute of Ageing, Shatin, Hong Kong; ^3^Division of Geriatric Medicine, Saint Louis University School of Medicine, St Louis, MO, United States; ^4^The Chinese University of Hong Kong Jockey Club Centre for Osteoporosis Care and Control, Shatin, Hong Kong

**Keywords:** cognitive frailty, cognitive impairment, frailty, length of hospital stay, mortality, physical limitation

## Abstract

**Objectives:**

To examine how various combinations of cognitive impairment (overall performance and specific domains) and pre-frailty predict risks of adverse outcomes; and to determine whether cognitive frailty may be defined as the combination of cognitive impairment and the presence of pre-frailty.

**Design:**

Community-based cohort study.

**Participants:**

Chinese men and women (*n* = 3,491) aged 65+ without dementia, Parkinson’s disease and/or frailty at baseline.

**Measurements:**

Frailty was characterized using the Cardiovascular Health Study criteria. Overall cognitive impairment was defined by a Cantonese Mini-Mental Status Examination (CMMSE) total score (<21/24/27, depending on participants’ educational levels); delayed recall impairment by a CMMSE delayed recall score (<3); and language and praxis impairment by a CMMSE language and praxis score (<9). Adverse outcomes included poor quality of life, physical limitation, increased cumulative hospital stay, and mortality.

**Results:**

Compared to those who were robust and cognitively intact at baseline, those who were robust but cognitively impaired were more likely to develop pre-frailty/frailty after 4 years (*P* < 0.01). Compared to participants who were robust and cognitively intact at baseline, those who were pre-frail and with overall cognitive impairment had lower grip strength (*P* < 0.05), lower gait speed (*P* < 0.01), poorer lower limb strength (*P* < 0.01), and poorer delayed recall at year 4 [OR, 1.6; 95% confidence interval (CI), 1.2–2.3]. They were also associated with increased risks of poor quality of life (OR, 1.5; 95% CI, 1.1–2.2) and incident physical limitation at year 4 (OR, 1.8; 95% CI, 1.3–2.5), increased cumulative hospital stay at year 7 (OR, 1.5; 95% CI, 1.1–2.1), and mortality over an average of 12 years (OR, 1.5; 95% CI, 1.0–2.1) after adjustment for covariates. There was no significant difference in risks of adverse outcomes between participants who were pre-frail, with/without cognitive impairment at baseline. Similar results were obtained with delayed recall and language and praxis impairments.

**Conclusion:**

Robust and cognitively impaired participants had higher risks of becoming pre-frail/frail over 4 years compared with those with normal cognition. Cognitive impairment characterized by the CMMSE overall score or its individual domain score improved the predictive power of pre-frailty for poor quality of life, incident physical limitation, increased cumulative hospital stay, and mortality. Our findings support to the concept that cognitive frailty may be defined as the occurrence of both cognitive impairment and pre-frailty, not necessarily progressing to dementia.

## Introduction

Frailty represents a state of decline in functional reserves, which increases the risk of adverse health outcomes such as morbidity, disability, and institutionalization, after a stressor event (1). It can be preceded by, but also occurs in the absence of chronic disease (2) and has been suggested as a better predictor of health and well-being than the presence or absence of disease. Although the term frailty is commonly used in clinical practice, there is no consensus on the definition of frailty. A popular approach to the assessment of frailty as proposed by Fried et al. (1) (i.e., the phenotype approach) encompasses the assessment of five criteria-based primarily on physical attributes and capabilities including poor grip strength, slow walking speed, low levels of physical activity, exhaustion, and unintentional weight loss, whereas an individual is considered to be frail if they present with three or more of five criteria. Another notable approach to the assessment of frailty is that of Rockwood and Mitnitski (3, 4) (i.e., the deficit accumulation model) in which frailty is viewed in terms of the number of health deficits (i.e., integration with measures of physical frailty and other domains) that are manifest in the individual, leading to a continuous measure of frailty (frailty index).

More recently, there is general consensus that measures of cognitive function should be added to physical performance for the definition of frailty, in that there is a bidirectional relationship between physical frailty and cognitive impairment. There is also a parallel pathway among frailty discourse, that cognitive vulnerability (or impairment) may be a precursor of mild neurodegenerative disorder [akin to pre-dementia state of Mild Cognitive Impairment (MCI)] (5) and subsequently major neurodegenerative disorder (dementia) (6). Numerous studies have demonstrated that cognitive impairment may lead to increased risk of acquiring individual components of frailty syndrome (e.g., faster gait speed decline)/future frailty (7–9). The reciprocal relationship, which frailty predicts cognitive decline/incident dementia, has also been reported (10–14). Both frailty and cognitive impairment share many common risk factors and underlying mechanisms (6, 15, 16). Although many studies demonstrate close relationship between frailty and cognitive impairment, most of them have characterized frailty and cognitive impairment as two different entities, and the term “cognitive frailty” has been proposed, to characterize the co-existence of both frailty and cognitive impairment. An international consensus group organized by the International Academy on Nutrition and Aging (IANA) and the International Association of Gerontology and Geriatrics (IAGG) proposed the definition as a clinical condition characterized by the simultaneous presence of both physical frailty and MCI (Clinical Dementia Rating = 0.5) (17). Recent studies have reported that cognitive frailty conferred additional greater risk of adverse outcomes including disability, hospitalization, and mortality (6, 18–20). Understanding the temporal relationship between cognitive impairment and frailty is important, in predicting the onset of the other, with implications for screening and intervention programs. For example, in the Baltimore longitudinal study of aging, a bidirectional relationship was noted for usual gait speed and executive function, with each predicting change in the other, while poor fast walking performance predicted future executive function and memory changes but not *vice versa* (14). Although there is no universal consensus regarding the entity of cognitive frailty and its definition, there is general consensus of the importance of recognizing cognitive impairment, as differentiated from screening for dementia (21).

According to the IANA/IAGG, the primary criterion of cognitive frailty is the presence of physical frailty and MCI, without dementia. However, different states of cognitive vulnerability and frailty may be relevant to identify older persons with cognitive frailty. Furthermore, it is likely that MCI may represent a later stage of cognitive impairment at which multiple domains of cognition have already occurred. Early detection of abnormalities in specific domains of cognitive function (e.g., memory problems, difficulties in word finding) together with identification of the pre-frail state (an intermediate stage between non-frail and frail) may allow opportunities for reversibility through intervention strategies, which is supported by the findings from a home-based program to prevent functional decline in physically frail elderly persons in which the benefit of the program was observed among those with moderate frailty, but not those with severe frailty (22). Using the Mr and MsOs study of older Chinese men and women who were free of dementia and/or Parkinson’s disease and who were non-frail at baseline, we examined how various combinations of cognitive impairment (overall performance as well as two selected *a priori* domains) and pre-frailty predict risks of adverse outcomes (poor quality of life, physical limitation, increased cumulative hospital stay, and mortality), and to determine whether cognitive frailty may be defined as the combination of cognitive impairment (overall or domain specific) and the presence of pre-frailty.

## Materials and Methods

### Participants

**Figure 1:**
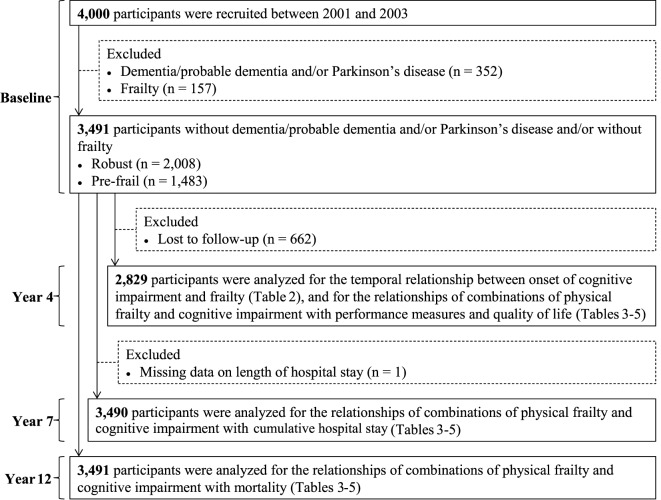
Flow chart of study participants included in the respective analysis.

Four thousand community-dwelling Chinese men and women aged 65 years and older were recruited for a cohort study on osteoporosis and general health (Mr and MsOs study) in Hong Kong between August 2001 and December 2003 by placing recruitment notices in housing estates and community centers for older adults. Several talks were also given at these centers explaining the purpose, procedures, and investigations to be carried out. Participants were volunteers, and the aim was to recruit a stratified sample so that approximately 33% would each be aged 65–69, 70–74, and 75 years and older. Those who were unable to walk independently, had bilateral hip replacement, or were not competent to give informed consent were excluded. Eligible persons were invited to attend a health check at the School of Public Health, The Chinese University of Hong Kong. A team of trained research assistants administered the study questionnaire and took physical measurements for each participant on the same day. In the present study, we excluded 352 participants who had reported a history of dementia/probable dementia [Cantonese Mini-Mental Status Examination (CMMSE) total score <18 (no education), <21 (primary school), or <25 (secondary school and above)] and/or Parkinson’s disease and 157 participants who were frail at baseline, yielding a study of 3,491 participants for the descriptive analysis. Participants (*n* = 662) who did not assess for frailty at the 4-year follow-up were further excluded from the analyses for the risk prediction of adverse outcomes. The valid study population included in the respective analysis is shown in Figure 1. Details of the study population have been reported elsewhere (23). All participants gave written consent in accordance with the Declaration of Helsinki. The study was approved by the Clinical Research Ethics Committee of the Chinese University of Hong Kong (CRE-2003.102).

### Questionnaire

The information from the questionnaire used in this study included demographics, educational levels, socioeconomic status, smoking habits, alcohol intake, physical activity, quality of diet, quality of life, and daily functioning. Socioeconomic status was assessed by asking the participants to mark their self-perceived position on a ladder with 10 rungs, with the lowest and highest rungs representing the lowest and highest socioeconomic status in society (Hong Kong ladder). Smoking habits were categorized as non-current smoker and current smoker. Alcohol intake was categorized as non-drinker (≤12 alcoholic drinks in the past 12 months) and drinker (>12 alcoholic drinks in the past 12 months). Physical activity levels were assessed using the Physical Activity Scale of the Elderly (PASE) (24). The quality of diet was assessed using the Diet Quality Index-International (DQI-I) (25). Quality of life was assessed using the 12-Item Short Form Health Survey (SF-12) (26). Information on daily functioning was obtained regarding impairment in walking two to three blocks outside on level ground, climbing up 10 steps without resting, preparing own meals, doing heavy housework, such as scrubbing floors or washing windows, and doing own shopping for groceries or clothes.

### Physical Measurements

Body weight was measured with the Physician Balance Beam Scale (Health-O-Meter, Arlington Heights, IL, USA). Height was measured with the Holtain Harpenden stadiometer (Holtain, Crosswell, UK). Body mass index (BMI) was calculated by dividing the weight in kilogram by height in meter squared. Grip strength was measured using a dynamometer (JAMAR Hand Dynamometer 5030JO; Sammons Preston, Bolingbrook, IL, USA). Two readings were taken from each side and the maximum value of the right or left was used for analysis. The intra-class correlation coefficients for right and left handgrip strength were 0.921 [95% confidence interval (CI), 0.914–0.927] and 0.916 (95% CI, 0.909–0.923), respectively. Gait speed was measured using the best time in seconds to complete a walk along a straight line 6 m long in distance. A warm up period of less than 5 min was followed by two walks, and the best time was recorded. The intra-class correlation coefficient for the two walking trials was 0.752 (95% CI, 0.732–0.770). Chair stand was measured by asking the participant to rise from a chair (seat height 45 cm), with arms folded across the chest, five times as quickly as possible. The time taken was recorded.

### Frailty Assessment

Frailty was assessed using the five-item Cardiovascular Health Study (CHS) frailty phenotype, with total score ranging from 0 to 5 (1). The five items are unintentional weight loss, self-rated exhaustion, weakness (grip strength), slow walking speed, and low physical activity. The equivalent variables used in this study for the construction of the CHS score were BMI less than 18.5 kg/m^2^, having no energy, grip strength measurement in the lowest quartile, walking speed measurement in the lowest quartile, and PASE score in the lowest quartile. The total scores were used to categorize participants as robust (score = 0), pre-frail (score = 1–2), and frail (score = 3–5).

### Cognitive Function Assessment

Cognitive function was assessed using the CMMSE (27). CMMSE is a validated Cantonese version of Mini-Mental Status Examination (28), which is composed of 30 items that assess multiple domains of cognitive function, including tests of orientation to time (max score: 5) and place (max score: 5), registration (max score: 3), attention and calculation (max score: 5), recall (max score: 3) and language and praxis (max score: 9). Score by the CMMSE is ranged from 0 to 30; a lower CMMSE score reflects more dementia-related cognitive impairment. A score of less than 21 in individuals with no education, a score of less than 24 in individuals with primary education, or a score of less than 27 in well-educated individuals with secondary or tertiary education are identified as overall cognitive impairment. Alternatively, individual who failed to recall any of the three words during the CMMSE delayed recall (i.e., a CMMSE delayed recall score of less than 3) or were unable to complete one or more language and praxis tasks on the CMMSE (i.e., a CMMSE language and praxis score of less than 9) were classified as cognitive impairment. These two domains (one amnestic and one non-amnestic) were selected *a priori*.

### Adverse Outcomes at Follow-up

Participants were invited to return for re-assessments after 4 years. Quality of life was assessed using the SF-12. Physical limitation was assessed using the following two questions: do you have any difficulty in climbing stairs (possible answers: no, a little, a lot) and do you have any difficulty in carrying out the following household activities such as moving chairs or tables (possible answers: no, a little, a lot). Participants were categorized as having physical limitation if the answer to either question was “a little” or “a lot,” while those who answered “no” to both questions were categorized as having no physical limitation. Incident physical limitation was defined as progression from those without limitation at baseline to having limitation at follow-up. Cumulative length of hospital stay from baseline to year 7 was obtained from the Hong Kong Hospital Authority records, which covered more than 93% of the hospitalizations in the Hong Kong population. The cutoff date for determining length of hospital stay was 30 September 2008. Increased cumulative hospital stay refers to the highest quintile (i.e., 20 days). Mortality was documented through a search of the Hong Kong Death Registry. The cutoff date for determining mortality was 31 March 2014.

### Data Analysis

Data were summarized as means (SDs) for continuous variables and as percentages for categorical data. Chi-square tests were used to compare the differences in the development of pre-frailty/frailty between robust/pre-frail participants with and without cognitive impairment at baseline. Analysis of covariance or logistic regression were performed to estimate the performance measures and the risk of adverse outcomes (poor quality of life, incident physical limitation, increased cumulative hospital stay, and mortality) after 4–12 years across groups of participants with different frailty (as per CHS criteria) and cognitive (as per CMMSE criteria) status at baseline, including (1) robust and cognitively intact, (2) robust and cognitively impaired, (3) pre-frail and cognitively intact, and (4) pre-frail and cognitively impaired. Covariates including age, sex, educational level, socioeconomic status, smoking habit, alcohol intake, physical activity, DQI-I, BMI, and baseline values of respective outcome variable were adjusted. The above analyses were repeated, substituting the CMMSE individual domain scores (delayed recall score, language and praxis score) in place of the CMMSE total score. All analyses were carried out using the Window-based SPSS Statistical Package (v23.0; SPSS, Inc., Chicago, IL, USA), and *P* values less than 0.05 were considered statistically significant.

## Results

At baseline, the mean age of the study sample was 72.0 (4.9) years, 48.4% were female, and 72.0% had primary or lower education. In total, 57.5% were robust, 42.5% were pre-frail, and 17.4% had overall cognitive impairment, with their CMMSE total score <21–27 (depending on participants’ educational levels). Of those who were pre-frail (*n* = 1,483), 20.4% had overall cognitive impairment (CMMSE total score < 21–27), 55.3% had delayed recall impairment (delayed recall score < 3), and 51.4% had language and praxis impairment (language and praxis score < 9) (Table 1).

**Table 1:** Descriptive characteristic of participants at baseline.

Baseline characteristic	Mean ± SD/*n*(%)
All participants	
Age, years	72.03 ± 4.91
Sex	
Male	1,800 (51.56)
Female	1,691 (48.44)

Educational levels	
No education	721 (20.65)
Primary school	1,793 (51.36)
Secondary school or above	977 (27.99)

Social economic status ladder—Hong Kong^a^	
≤4	1,411 (42.51)
>4	1,908 (57.49)

Smoking habits	
Current smokers	243 (6.95)
Non-current smokers	3,248 (93.04)

Alcohol intake^a^	
>12 alcoholic drinks in past 12 months	489 (14.01)
≤12 alcoholic drinks in past 12 months	3,001 (85.99)
Physical activity (PASE total score)	94.80 ± 42.87
Dietary intakes (DQI-I)^a^	64.71 ± 9.36
BMI, kg/m^2^	23.77 ± 3.22

Frailty	
Robust	2,008 (57.52)
Pre-frailty	1,483 (42.48)

Cognitive impairment	
Defined by CMMSE total score <21/24/27^b^	
No cognitive impairment	2,884 (82.61)
Cognitive impairment	607 (17.39)
Defined by CMMSE delayed recall score <3	
No cognitive impairment	1,592 (45.60)
Cognitive impairment	1,899 (54.40)
Defined by CMMSE language, repetition and commands score <9	
No cognitive impairment	1,811 (51.88)
Cognitive impairment	1,680 (48.12)

Participants with pre-frailty	
Among participants with pre-frailty	
W/o cognitive impairment (CMMSE total score ≥ 21/24/27)	1,181 (79.64)
W cognitive impairment (CMMSE total score < 21/24/27)	302 (20.36)

Among participants with pre-frailty	
W/o cognitive impairment (CMMSE delayed recall score = 3)	663 (44.71)
W cognitive impairment (CMMSE delayed recall score < 3)	820 (55.29)

Among participants with pre-frailty	
W/o cognitive impairment (CMMSE language and praxis score = 9)	721 (48.62)
W cognitive impairment (CMMSE language and praxis score < 9)	762 (51.38)

*^a^Missing observations (social economic status ladder—Hong Kong, *n* = 172; alcohol intake, *n* = 1; DQI-I, *n* = 4)*.

*^b^Cognitive impairment was defined by CMMSE total score <21 (no education), <24 (primary school), or <27 (secondary school and above)*.

*CMMSE, Cantonese Mini-Mental Status Examination; DQI-I, Diet Quality Index-International; PASE, Physical Activity Scale of the Elderly; BMI, body mass index*.

The prevalence of overall cognitive impairment was higher in the pre-frail group (17.8%) than in the robust group (14.4%). Compared to participants who were robust and cognitively intact at baseline, those who were robust but cognitively impaired were more likely to develop pre-frailty/frailty after 4 years (*P* < 0.01). Participants who were pre-frail but cognitively intact at baseline were also more likely to develop frailty at the 4-year follow-up than their cognitively impaired counterparts. However, the association was not significant (*P* = 0.056) (Table 2).

**Table 2:** Transitions in frailty status over 4 years by cognitive status according to baseline CMMSE total score.

	Cognitive impairment at baseline^a^		
	No	Yes	
	(*n* = 2,383)	(*n* = 446)	*P*
Participants who were robust at baseline and were reassessed at year 4	1,460	246	
Robust at baseline and follow-up	818 (56.03)	115 (46.75)	
Robust at baseline and pre-frail at follow-up	604 (41.37)	120 (48.78)	
Robust at baseline and frail at follow-up	38 (2.60)	11 (4.47)	0.007^†^
Participants who were pre-frailty at baseline and were reassessed at year 4	923	200	
Pre-frail at baseline and robust at follow-up	274 (29.69)	48 (24.00)	
Pre-frail at baseline and follow-up	535 (57.96)	135 (67.50)	
Pre-frail at baseline and frail at follow-up	114 (12.35)	17 (8.50)	0.056^‡^

*Data are reported as either number (percentage)*.

*^a^Cognitive impairment was defined by Cantonese Mini-Mental Status Examination total score <21 (no education), <24 (primary school), or <27 (secondary school and above)*.

*^†^*P*-value was obtained from Chi-square test comparing the differences in the development of frailty (with pre-frail and frail participants collapsed into one group) between robust participants with and without cognitive impairment at baseline*.

*^‡^P-value was obtained from Chi-square test comparing the differences in the development of frailty between pre-frail participants with and without cognitive impairment at baseline. Participants who were pre-frail at baseline and robust at follow-up were excluded*.

Compared to participants who were robust and cognitively intact at baseline, those who were pre-frail and with overall cognitive impairment had lower grip strength (*P* < 0.05), lower gait speed (*P* < 0.01), poorer lower limb strength (*P* < 0.01), and poorer performance in delayed recall at year 4 (OR, 1.6; 95% CI 1.2–2.3). They were also associated with increased risks of poor quality of life (OR, 1.5; 95% CI, 1.1–2.2) and incident physical limitation at year 4 (OR, 1.8; 95% CI, 1.3–2.5), increased cumulative hospital stay at year 7 (OR, 1.5; 95% CI, 1.1–2.1), and mortality over an average of 12 years (OR, 1.5; 95% CI, 1.0–2.1) after adjustment for covariates. Participants who were pre-frail and cognitively impaired at baseline were also associated with a higher risk of incident physical limitation at year 4 (OR, 1.8; 95% CI, 1.1–2.8) as compared to the robust but cognitively impaired participants, and had poorer cognitive performance at year 4 as compared to their cognitively intact counterparts (*P* < 0.01). However, there was no significant difference in risks of adverse outcomes between participants who were pre-frail, with or without cognitive impairment at baseline (Table 3).

**Table 3:** Performance measures, quality of life, and risk of adverse outcomes of participants in different frailty and cognitive status according to baseline CMMSE total score.^a^

	Robust		Pre-frailty		*P*/OR(95% CI)^†^		
Outcome	No cognitive impairment^(1)^ (*n* = 1,703)	Cognitive impairment^(2)^ (*n* = 305)	No cognitive impairment^(3)^ (*n* = 1,181)	Cognitive impairment^(4)^ (*n* = 302)	(1 vs. 4)	(2 vs. 4)	(3 vs. 4)
**Physical performance at year 4^b^**							
Grip strength, kg	28.55 ± 7.99	24.96 ± 7.28	25.20 ± 7.69	22.35 ± 7.03	0.013	0.749	0.286
Gait speed, m/s	1.00 ± 0.21	0.95 ± 0.21	0.88 ± 0.23	0.84 ± 0.22	0.002	0.719	0.138
Five chair stand, s	9.75 ± 3.56	10.16 ± 3.80	11.60 ± 5.87	12.20 ± 6.27	0.001	0.118	0.729

**Cognitive performance at year 4^b^**							
Global cognitive functioning							
CMMSE total score	26.89 ± 2.95	24.61 ± 3.95	26.31 ± 3.20	24.93 ± 4.06	0.063	0.437	0.006
Domain-specific cognition							
CMMSE time orientation score <5	251 (17.19)	72 (29.27)	203 (21.99)	56 (28.00)	1.09 (0.72, 1.64)	0.88 (0.53, 1.45)	1.04 (0.69, 1.56)
CMMSE place orientation score <5	333 (22.81)	80 (32.52)	280 (30.34)	69 (34.50)	1.11 (0.76, 1.62)	1.08 (0.68, 1.71)	0.99 (0.68, 1.43)
CMMSE registration score <3	35 (2.40)	14 (5.69)	37 (4.01)	9 (4.50)	1.55 (0.65, 3.68)	0.91 (0.34, 2.44)	1.35 (0.62, 2.96)
CMMSE attention/calculation score <5	572 (39.18)	156 (63.41)	401 (43.45)	115 (57.50)	1.04 (0.71, 1.54)	0.70 (0.44, 1.13)	0.91 (0.61, 1.36)
CMMSE delayed recall score <3	537 (36.78)	117 (47.56)	373 (40.41)	104 (52.00)	1.63 (1.17, 2.28)	1.39 (0.91, 2.14)	1.54 (1.10, 2.17)
CMMSE language and praxis score <9	780 (53.42)	166 (67.48)	573 (62.08)	134 (67.00)	1.33 (0.92, 1.92)	1.05 (0.66, 1.66)	1.08 (0.75, 1.57)

**Adverse outcomes at year 4–12^b^**							
Poor quality of life (SF-12 PCS) at year 4	328 (22.47)	60 (24.39)	302 (32.72)	70 (35.00)	1.53 (1.06, 2.22)	1.39 (0.84, 2.29)	1.09 (0.76, 1.57)
Poor quality of life (SF-12 MCS) at year 4	279 (19.11)	46 (18.70)	207 (22.43)	58 (29.00)	1.28 (0.86, 1.91)	1.29 (0.75, 2.21)	1.33 (0.90, 1.95)
Incident physical limitation at year 4	374 (25.62)	76 (30.89)	332 (35.97)	86 (43.00)	1.78 (1.26, 2.51)	1.78 (1.13, 2.82)	1.23 (0.87, 1.72)
Increased cumulative hospital stay at year 7	278 (16.32)	49 (16.12)	306 (25.91)	75 (24.83)	1.48 (1.06, 2.06)	1.53 (0.96, 2.44)	1.06 (0.77, 1.46)
Mortality over an average of 12 years	232 (13.62)	44 (14.43)	261 (22.10)	71 (23.51)	1.46 (1.02, 2.07)	1.55 (0.94, 2.54)	1.19 (0.85, 1.67)

*Data are reported as either number (percentage) or mean ± SD*.

*^a^Cognitive impairment was defined by CMMSE total score <21 (no education), <24 (primary school), or <27 (secondary school and above)*.

*^b^Analyses were based on valid cases observed for grip strength (*n* = 2,798), gait speed (*n* = 2,821), five chair stand (*n* = 2,789), CMMSE, SF-12 and incident physical limitation (*n* = 2,829), increased cumulative hospital stay (*n* = 3,490), and mortality (*n* = 3,491)*.

*^†^*P*-values/ORs (95% CI) were obtained from multivariate linear regression/logistic regression adjusting for age, sex, education (below secondary vs. secondary or above), social economic status ladder—Hong Kong (≤4 vs. >4), smoking (current smokers vs. non-current smokers), alcohol intake (>12 vs. ≤12 alcoholic drinks in past 12 m), physical activity (PASE total score), dietary intakes (DQI-I), BMI, and baseline value of respective outcome variable (when appropriate)*.

*CMMSE, Cantonese Mini-Mental Status Examination; DQI-I, Diet Quality Index-International; PASE, Physical Activity Scale of the Elderly; BMI, body mass index*.

When a single *a priori* selected cognitive domain was used to define cognitive impairment, participants with pre-frailty and a delayed recall score <3 at baseline had lower gait speed (*P* < 0.001), poorer lower limb strength (*P* < 0.05), poorer cognitive performance in terms of time orientation (OR, 1.7; 95% CI 1.3–2.3), place orientation (OR, 1.7; 95% CI, 1.3–2.2), attention/calculation (OR, 1.5; 95% CI, 1.1–1.9), as well as language and praxis at year 4 (OR, 1.5; 95% CI, 1.1–1.9). They were also associated with increased risks of poor quality of life (OR, 1.7; 95% CI, 1.3–2.3), and incident physical limitation at year 4 (OR, 1.8; 95% CI, 1.4–2.3), and increased cumulative hospital stay at year 7 (OR, 1.4; 95% CI, 1.1–1.9) as compared to participants who were robust and had a delayed recall score = 3 (Table 4). Similar results were obtained when cognitive impairment was redefined by language and praxis score (Table 5).

**Table 4:** Performance measures, quality of life, and risk of adverse outcomes of participants in different frailty and cognitive status according to baseline CMMSE delayed recall score.^a^

	Robust		Pre-frailty		*P*/OR(95% CI)^†^		
Outcome	No cognitive impairment^(1)^ (*n* = 929)	Cognitive impairment^(2)^ (*n* = 1,079)	No cognitive impairment^(3)^ (*n* = 663)	Cognitive impairment^(4)^ (*n* = 820)	(1 vs. 4)	(2 vs. 4)	(3 vs. 4)
**Physical performance at year 4^b^**							
Grip strength, kg	28.47 ± 7.92	27.65 ± 8.03	25.33 ± 7.92	24.17 ± 7.39	0.098	0.010	0.681
Gait speed, m/s	1.01 ± 0.21	0.97 ± 0.21	0.89 ± 0.23	0.86 ± 0.22	<0.001	0.085	0.543
Five chair stand, s	9.61 ± 3.51	9.98 ± 3.67	11.50 ± 5.65	11.88 ± 6.18	0.027	0.015	0.323

**Cognitive performance at year 4^b^**							
Global cognitive functioning							
CMMSE total score	27.06 ± 2.84	26.13 ± 3.45	26.57 ± 3.08	25.66 ± 3.60	0.114	0.255	0.536
Domain-specific cognition							
CMMSE time orientation score <5	128 (16.10)	195 (21.41)	94 (18.58)	165 (26.74)	1.71 (1.26, 2.33)	1.40 (1.06, 1.86)	1.48 (1.09, 2.01)
CMMSE place orientation score <5	154 (19.37)	259 (28.43)	145 (28.66)	204 (33.06)	1.66 (1.25, 2.21)	1.07 (0.83, 1.38)	1.08 (0.82, 1.42)
CMMSE registration score <3	20 (2.52)	29 (3.18)	20 (3.95)	26 (4.21)	1.45 (0.69, 3.04)	1.15 (0.61, 2.15)	1.02 (0.54, 1.93)
CMMSE attention/calculation score <5	317 (39.87)	411 (45.12)	213 (42.09)	303 (49.11)	1.46 (1.12, 1.90)	1.24 (0.96, 1.59)	1.39 (1.07, 1.81)
CMMSE delayed recall score <3	235 (29.56)	419 (45.99)	163 (32.21)	314 (50.89)	1.21 (0.75, 1.96)	1.05 (0.83, 1.32)	1.11 (0.68, 1.79)
CMMSE language and praxis score <9	414 (52.08)	532 (58.40)	303 (59.88)	404 (65.48)	1.46 (1.13, 1.89)	1.31 (1.02, 1.67)	1.22 (0.94, 1.58)

**Adverse outcomes at year 4–12^b^**							
Poor quality of life (SF-12 PCS) at year 4	162 (20.38)	226 (24.81)	159 (31.42)	213 (34.52)	1.68 (1.25, 2.26)	1.34 (1.03, 1.75)	1.12 (0.85, 1.47)
Poor quality of life (SF-12 MCS) at year 4	132 (16.60)	193 (21.19)	116 (22.92)	149 (24.15)	1.35 (0.98, 1.85)	0.89 (0.66, 1.19)	1.05 (0.78, 1.42)
Incident physical limitation at year 4	187 (23.52)	263 (28.87)	181 (35.77)	237 (38.41)	1.78 (1.36, 2.34)	1.48 (1.15, 1.91)	1.10 (0.85, 1.43)
Increased cumulative hospital stay at year 7	136 (14.66)	191 (17.70)	174 (26.24)	207 (25.24)	1.43 (1.08, 1.91)	1.20 (0.93, 1.55)	0.83 (0.65, 1.08)
Mortality over an average of 12 years	125 (13.46)	151 (13.99)	138 (20.81)	194 (23.66)	1.27 (0.94, 1.72)	1.17 (0.88, 1.55)	1.01 (0.76, 1.32)

*Data are reported as either number (percentage) or mean ± SD*.

*^a^Cognitive impairment was defined by a CMMSE delayed recall score of <3*.

*^b^Analyses were based on valid cases observed for grip strength (*n* = 2,798), gait speed (*n* = 2,821), five chair stand (*n* = 2,789), CMMSE, SF-12 and incident physical limitation (*n* = 2,829), increased cumulative hospital stay (*n* = 3,490), and mortality (*n* = 3,491)*.

*^†^*P*-values/ORs (95% CI) were obtained from multivariate linear regression/logistic regression adjusting for age, sex, education (below secondary vs. secondary or above), social economic status ladder—Hong Kong (≤4 vs. >4), smoking (current smokers vs. non-current smokers), alcohol intake (>12 vs. ≤12 alcoholic drinks in past 12 m), physical activity (PASE total score), dietary intakes (DQI-I), BMI, and baseline value of respective outcome variable (when appropriate)*.

*CMMSE, Cantonese Mini-Mental Status Examination; DQI-I, Diet Quality Index-International; PASE, Physical Activity Scale of the Elderly; BMI, body mass index*.

**Table 5:** Performance measures, quality of life, and risk of adverse outcomes of participants in different frailty and cognitive status according to baseline CMMSE language and praxis score.^a^

	Robust		Pre-frailty		*P/*OR(95% CI)^†^		
Outcome	No cognitive impairment^(1)^ (*n* = 1,090)	Cognitive impairment^(2)^ (*n* = 918)	No cognitive impairment^(3)^ (*n* = 721)	Cognitive impairment^(4)^ (*n* = 762)	(1 vs. 4)	(2 vs. 4)	(3 vs. 4)
**Physical performance at year 4^b^**							
Grip strength, kg	29.51 ± 8.22	26.20 ± 7.28	26.05 ± 7.80	23.36 ± 7.27	0.094	0.069	0.944
Gait speed, m/s	1.02 ± 0.21	0.95 ± 0.21	0.91 ± 0.23	0.83 ± 0.21	<0.001	0.037	0.038
Five chair stand, s	9.42 ± 3.19	10.29 ± 3.99	11.06 ± 4.95	12.35 ± 6.72	0.001	0.246	0.901

**Cognitive performance at year 4^b^**							
Global cognitive functioning							
CMMSE total score	27.32 ± 2.74	25.63 ± 3.50	26.83 ± 3.11	25.32 ± 3.52	0.012	0.532	0.851
Domain-specific cognition							
CMMSE time orientation score <5	131 (13.88)	192 (25.20)	98 (17.66)	161 (28.35)	2.08 (1.51, 2.88)	1.22 (0.91, 1.64)	1.28 (0.94, 1.76)
CMMSE place orientation score <5	211 (22.35)	202 (26.51)	159 (28.65)	190 (33.45)	1.30 (0.98, 1.73)	1.17 (0.88, 1.54)	0.94 (0.71, 1.25)
CMMSE registration score <3	13 (1.38)	36 (4.72)	22 (3.96)	24 (4.23)	3.51 (1.49, 8.27)	0.83 (0.44, 1.55)	0.88 (0.46, 1.68)
CMMSE attention/calculation score <5	325 (34.43)	403 (52.89)	214 (38.56)	302 (53.17)	1.63 (1.24, 2.12)	1.08 (0.82, 1.41)	1.28 (0.98, 1.68)
CMMSE delayed recall score <3	326 (34.53)	328 (43.04)	211 (39.82)	256 (45.07)	1.55 (1.19, 2.01)	1.02 (0.79, 1.32)	1.21 (0.93, 1.56)
CMMSE language and praxis score <9	434 (45.97)	512 (67.19)	296 (53.33)	411 (72.36)	2.41 (1.53, 3.80)	1.14 (0.87, 1.50)	1.70 (1.07, 2.69)

**Adverse outcomes at year 4–12^b^**							
Poor quality of life (SF-12 PCS) at year 4	204 (21.61)	184 (24.15)	152 (27.39)	220 (38.73)	1.82 (1.36, 2.42)	1.69 (1.28, 2.24)	1.65 (1.24, 2.19)
Poor quality of life (SF-12 MCS) at year 4	175 (18.54)	150 (19.69)	118 (21.26)	147 (25.88)	1.08 (0.79, 1.49)	1.36 (1.00, 1.86)	1.19 (0.87, 1.63)
Incident physical limitation at year 4	215 (22.78)	235 (30.84)	178 (32.07)	240 (42.25)	1.91 (1.46, 2.51)	1.65 (1.27, 2.15)	1.30 (0.99, 1.71)
Increased cumulative hospital stay at year 7	166 (15.23)	161 (17.56)	184 (25.52)	197 (25.85)	1.46 (1.09, 1.94)	1.32 (1.00, 1.74)	0.89 (0.68, 1.16)
Mortality over an average of 12 years	153 (14.04)	123 (13.40)	161 (22.33)	171 (22.44)	1.14 (0.83, 1.55)	1.29 (0.95, 1.76)	0.83 (0.62, 1.10)

*Data are reported as either number (percentage) or mean ± SD*.

*^a^Cognitive impairment was defined by a CMMSE language and praxis score of <9*.

*^b^Analyses were based on valid cases observed for grip strength (*n* = 2,798), gait speed (*n* = 2,821), five chair stand (*n* = 2,789), CMMSE, SF-12 and incident physical limitation (*n* = 2,829), increased cumulative hospital stay (*n* = 3,490), and mortality (*n* = 3,491)*.

*^†^*P*-values/ORs (95% CI) were obtained from multivariate linear regression/logistic regression adjusting for age, sex, education (below secondary vs. secondary or above), social economic status ladder—Hong Kong (≤4 vs. >4), smoking (current smokers vs. non-current smokers), alcohol intake (>12 vs. ≤12 alcoholic drinks in past 12 m), physical activity (PASE total score), dietary intakes (DQI-I), BMI, and baseline value of respective outcome variable (when appropriate)*.

*CMMSE, Cantonese Mini-Mental Status Examination; DQI-I, Diet Quality Index-International; PASE, Physical Activity Scale of the Elderly; BMI, body mass index*.

The risks of having adverse outcomes at follow-up were also compared between those who were robust and cognitively intact and the rest of the groups. As expected, participants who were pre-frail but cognitively intact at baseline were associated with increased risk of poor quality of life (OR, 1.4; 95% CI, 1.1–1.7) and incident physical limitation at year 4 (OR, 1.5; 95% CI, 1.2–1.8) as well as increased cumulative hospital stay at year 7 (OR, 1.4; 95% CI, 1.2–1.8) as compared to participants who were robust and cognitively intact at baseline. However, there was no significant difference in risks of adverse outcomes between participants who were robust, with or without cognitive impairment at baseline (Table S1 in Supplementary Material).

## Discussion

In a cohort of older people free of dementia and/or Parkinson’s disease and/or frailty at baseline, we showed that robust and cognitively impaired participants were more likely to develop pre-frailty/frailty after 4 years than the robust and cognitively intact participants. Furthermore, participants with both pre-frailty and cognitive impairment at baseline had poorer physical and cognitive performances, higher risks of poor quality of life, incident physical limitation, increased cumulative hospital stay, and mortality over follow-up than those with none of these conditions. These findings support a concept of the combination of cognitive impairment (overall or specific domains) and pre-frailty representing cognitive frailty, with subsequent adverse consequences. In view of the reversibility of the frailty continuum (29) and non-pharmacological strategies to improve frailty status and cognitive impairment (30–34), early detection of cognitive frailty has public health implications since participation in group exercises that combines aerobic and resistance elements with or without cognitive training may retard decline or even lead to some improvement (35). This concept of earlier detection of abnormalities is similar to the current thinking in dementia research, where intervention may be more effective if applied at an early stage.

Our finding is in close agreement with some previous studies which consistently show a higher prevalence of cognitive impairment among physically pre-frail/frail elderly (14, 36), supporting the notion that physical and cognitive impairment are closely related and are integral components of frailty. Our findings also extend a previous study examining the association of impaired cognition with frailty (8, 9) by showing the longitudinal relationship between low cognitive scores and higher risk of incident pre-frailty/frailty, which support results of previous studies proposing the inclusion of cognitive function in the assessment of frailty (3, 37, 38). Several mechanisms might explain the association between cognitive impairment and increased risk of frailty. First, poor cognition in robust individuals may be associated with underlying risk factors (e.g., poor nutritional status, physical inactivity) known to affect the development of frailty. Second, the association could reflect the existence of shared factors (e.g., increased inflammatory markers) that may be causing cognitive decline and the onset of frailty (39, 40).

Given the demonstrated increased risk of developing frailty associated with cognitive impairment at baseline, we further examined the physical and cognitive profile at the 4-year follow-up of participants with both cognitive impairment and pre-frailty at baseline. These participants had lower grip strength, lower gait speed, and poorer performance in the chair stand test as compared to robust and cognitively intact participants; and had poorer cognitive performance in the CMMSE test (in terms of CMMSE total score) compared to their cognitively intact counterparts. Furthermore, they had significantly lower delayed recall domain score. These findings concur with findings from a recent study, which demonstrated that individuals with cognitive frailty showed worse performance in cognitive function, as assessed by a battery of neuropsychological tests than their cognitively normal peers (41). However, these participants did not have poorer performance in non-memory function, suggesting that memory function may decline first in the pre-frail state, while non-memory cognitive function such as executive function and attention may be more closely associated with frailty, but not pre-frailty (42).

In the present study, participants with pre-frailty and cognitive impairment at baseline had increased risks of poor quality of life, incident physical limitation, increased cumulative hospital stay, and mortality over follow-up, independent of age, sex, educational levels, and other potential cofounders. These findings are compatible with previous findings that a measure of frailty that combines a range of diverse deficits, including cognitive functioning, is a better predictor of adverse health outcomes. For example, in the Three-City Study and the Singapore Longitudinal Ageing Studies (18, 20), including cognitive impairment to the operational criteria defining the frailty phenotype could increase its predictive validity with regard to adverse health outcomes. In a sample of community-dwelling Koreans aged 65 years and older, frail persons with cognitive impairment had a lower survival rate as compared to those non-frail and not cognitively impaired (43).

Although cognitive impairment improves predictive validity of frailty, there is no consensus on how cognitive impairment should be defined, and numerous different criteria exist (e.g., amnestic and non-amnestic cognitive impairment; single-domain and multiple-domain impairment). Cognitive impairment is a transitional state between normal cognition and dementia; thus, varying the threshold used for defining impairment would results in different rates of cognitive impairment. To capture cognitive impairment at a point at which the decline in multiple systems is still occurring in its earliest stages, the early symptoms of cognitive impairment (e.g., memory problems, difficulties in word finding) were tested against multiple-domain cognitive impairment to be used in the criteria for cognitive impairment in terms of their predictive value of adverse outcomes. Our findings demonstrated that lower scores on the two selected *a priori* domains (delayed recall as well as language and praxis) in combinations with pre-frailty at baseline were associated with higher risks of incident physical limitation and increased cumulative hospital stay over follow-up, suggesting that single-domain cognitive impairment may be useful in risk prediction. Although evidence has shown that multiple-domain amnestic cognitive impairment may be a better predictor of dementia than single-domain amnestic or non-amnestic cognitive impairment (44, 45), those with single-domain cognitive impairment have a relatively high rate of reversion to normal cognition (46). Furthermore, multiple-domain cognitive impairment possibly represents a heterogeneous group of individuals with different neuropsychological profiles; hence subtyping cognitive impairment according to number and types of domains impaired may improve the characterization of the cognitive impairment construct and be useful for risk prediction in relation to different outcomes (47). From the clinical practice point of view, a short screening tool would be important, followed by interventions. Our findings suggest that the use of single cognitive domain may be effective in characterizing cognitive impairment groups; and the use of pre-frailty also identifies a subset of individuals at risk of progressing to frailty. Taken together, the findings of this study together with current available literature of cross-sectional and longitudinal studies lend support to the concept that cognitive frailty may be defined as the existence of overall cognitive impairment (or an individual domain) together with pre-frailty. This definition obviates the need for a psychiatric diagnosis such as the concomitant diagnosis of MCI (as proposed by Kelaiditi et al.) (17), or the need to consider cognitive frailty as a precursor condition of dementia.

There were some limitations in this study. First, the study participation was voluntary which could result in selection bias. Compared to the general elderly population in Hong Kong, the participants may represent those who are more robust, as they tended to be more health conscious, had a higher educational level and more physical active. The other limitation relates to the use of CMMSE for delineation of cognitive status. Due to ceiling effect, it may under-diagnose individuals with early dementia such that these individuals were included in the sample. Similarly, it may under-diagnose cognitive impairment such that some individuals, in particular highly educated individuals, are classified as “no cognitive impairment,” (48) albeit the expected effect would be a bias toward the null. Another limitation is the use of *a priori* selected domains and the arbitrary domain scores from CMMSE, which would be expected to be less psychometrically robust compared with domain scores derived from a neuropsychological battery, and may potentially lead to more false-positives among older people with lower educational levels. Another limitation could be represented by the collection of length of hospital stay using the Hong Kong Hospital Authority records that do not cover the 100% of hospitalization in the Hong Kong population. Finally, the incidence of dementia was not available. However, data regarding incident dementia is being collected in an ongoing follow-up study, which allows incident dementia to be related to baseline cognitive frailty.

## Conclusion

In conclusion, our results showed that robust and cognitively impaired participants had higher risks of becoming pre-frail/frail over a period of 4 years than their counterparts with normal cognition. Furthermore, cognitive impairment improved the predictive power of pre-frailty for poor quality of life, incident physical limitation, increased cumulative hospital stay, and mortality. The findings of this study support to the concept that cognitive frailty may be defined as the occurrence of both cognitive impairment and pre-frailty [as opposed to established frailty as per the IANA/IAGG definition by Kelaiditi et al. (17)], not necessarily progressing to dementia. Our results also showed that lower scores in delayed recall as well as language and praxis, in combinations with pre-frailty, may also be used as criteria for cognitive impairment in terms of their predictive value of adverse outcomes.

## Ethics Statement

All participants gave written consent in accordance with the Declaration of Helsinki. The study was approved by the Clinical Research Ethics Committee of the Chinese University of Hong Kong.

## Author Contributions

RY, JM, TK, and JW conceived and designed the study and wrote the paper; RY, JL, and OC carried out the analysis.

## Conflict of Interest Statement

The authors declare that the research was conducted in the absence of any commercial or financial relationships that could be construed as a potential conflict of interest. The reviewer WL declared a past co-authorship with the authors JM, JW to the handling editor.
