# Supplementary material for: The Effects of Combinations of Cognitive Impairment and Pre-frailty on Adverse Outcomes from a Prospective Community-Based Cohort Study of Older Chinese People
Source: Front Med (Lausanne). 2018 Mar 6;5:50. doi: 10.3389/fmed.2018.00050 (PMC5863513; doi:10.3389/fmed.2018.00050)
Supplement: Supplementary file 1 [file table_1.DOCX]

**Supplementary table 1 Risk of adverse outcomes of participants in different frailty and cognitive status according to baseline CMMSE total score ^a^**

|  | **Robust** | | **Pre-frailty** | | **OR (95% CI)**^†^ | | |
| --- | --- | --- | --- | --- | --- | --- | --- |
| **Outcome** | **no cognitive**  **impairment ^(1)^** | **cognitive**  **impairment ^(2)^** | **no cognitive**  **impairment ^(3)^** | **cognitive**  **impairment ^(4)^** | (1 vs. 2) | (1 vs. 3) | (1 vs. 4) |
|  | (n = 1,703) | (n = 305) | (n = 1,181) | (n = 302) |  |  |  |
| ***Adverse outcomes at year 4-12***^b^ |  |  |  |  |  |  |  |
| Poor quality of life (SF-12 PCS) at year 4 | 328 (22.47) | 60 (24.39) | 302 (32.72) | 70 (35.00) | 1.09 (0.77, 1.55) | 1.40 (1.12, 1.74) | 1.53 (1.06, 2.22) |
| Poor quality of life (SF-12 MCS) at year 4 | 279 (19.11) | 46 (18.70) | 207 (22.43) | 58 (29.00) | 0.97 (0.66, 1.41) | 1.04 (0.82, 1.32) | 1.28 (0.86, 1.91) |
| Incident physical limitation at year 4 | 374 (25.62) | 76 (30.89) | 332 (35.97) | 86 (43.00) | 1.03 (0.74, 1.43) | 1.47 (1.19, 1.81) | 1.78 (1.26, 2.51) |
| Increased cumulative hospital stay at year 7 | 278 (16.32) | 49 (16.12) | 306 (25.91) | 75 (24.83) | 0.96 (0.67, 1.37) | 1.42 (1.15, 1.75) | 1.48 (1.06, 2.06) |
| Mortality over an average of 12 years | 232 (13.62) | 44 (14.43) | 261 (22.10) | 71 (23.51) | 1.00 (0.68, 1.47) | 1.18 (0.94, 1.49) | 1.46 (1.02, 2.07) |

Data are reported as either number (percentage) or OR (95% CI).

^a^Cognitive impairment was defined by CMMSE total score <21 (no education), <24 (primary school), or <27 (secondary school and above).

^b^Analyses were based on valid cases observed for SF-12 and incident physical limitation (n = 2,829), increased cumulative hospital stay (n = 3,490), and mortality (n = 3,491).

^†^OR (95%CI) were obtained from logistic regression adjusting for age, sex, educational levels (below secondary vs. secondary or above), social economic status ladder-Hong Kong (≤4 vs. >4), smoking (current smokers vs. non-current smokers), alcohol intake (>12 vs. ≤12 alcoholic drinks in past 12m), physical activity (PASE total score), dietary intakes (DQI-I), BMI, and baseline value of respective outcome variable (when appropriate).

*CMMSE, Cantonese Mini-Mental Status Examination; DQI-I, Diet Quality Index-International; PASE, Physical Activity Scale of the Elderly; BMI, body mass index.*
